# Daily Calcium and Vitamin D Supplementation as a Foundational Pillar in Osteoporosis Management: A Narrative Review

**DOI:** 10.3390/nu18142292

**Published:** 2026-07-13

**Authors:** María Pascual-Pastor, Mireia Barceló-Bru

**Affiliations:** Rheumatology Department, Vall d’Hebron University Hospital, 08035 Barcelona, Spain

**Keywords:** osteoporosis, calcium supplementation, vitamin D supplementation, fractures, antiresorptive treatment

## Abstract

Osteoporosis is a progressive skeletal disorder characterized by decreased bone strength and an increased risk of fractures. It presents a high global prevalence, thus imposing a significant clinical and economic burden. This narrative review includes current evidence on the role of combined calcium and vitamin D supplementation in the management and prevention of osteoporosis, as well as its effect on reducing fracture risk and maintaining bone mineral density. It aims to provide an overview of osteoporosis pathophysiology, the importance of adequate calcium levels and 25-hydroxyvitamin D concentrations and the prevalence of their deficiencies. The review examines the evidence supporting combined supplementation and its importance in patients receiving anti-osteoporotic pharmacological therapies. Current clinical guidelines and consensus statements, along with their recommendations for dosing, formulation, and monitoring of calcium and vitamin D supplements, are included. Barriers to the effective implementation, gaps in clinical practice, patient adherence, and primary care challenges are discussed. Ultimately, this review examines the cost-effectiveness of supplementation and the areas for improvement in real-world evidence and research in the field of osteoporosis. This work aims to provide clinicians and healthcare professionals with an updated, evidence-based framework for optimizing calcium and vitamin D supplementation in the care of patients with osteoporosis.

## 1. Introduction

Osteoporosis is a chronic, systemic, progressive skeletal disease characterized by decreased bone strength and increased susceptibility to fragility fractures. Bone strength is determined by both bone tissue quality and bone mineral density (BMD), and is compromised by microarchitectural deterioration that leads to increased skeletal fragility [1,2,3]. Although osteoporosis has traditionally been defined by BMD (T-score ≤ −2.5), nowadays this criterion is insufficient, as many fractures occur in individuals with higher BMD values [3]. For this reason, the National Bone Health Alliance has proposed extending the diagnosis to postmenopausal women and men ≥50 years of age with fragility fractures, regardless of BMD, or those with a high risk according to the Fracture Risk Assessment Tool (FRAX^®^) (≥20% risk of major fracture or ≥3% risk of hip fracture) [4]. Osteoporosis is classified into two types: primary and secondary. Primary osteoporosis is associated with menopause and ageing, whereas secondary osteoporosis is caused by systemic diseases, glucocorticoid consumption, lifestyle habits, or idiopathic causes [5,6]. Some of the risk factors of osteoporosis may be modifiable (smoking, alcohol consumption, sedentary lifestyle, and nutrition), while others are non-modifiable (age, sex, and genetics) [6,7,8].

Although osteoporosis is a common clinical disease in humans, age-related bone loss and skeletal changes similar to those seen in osteoporosis have also been described in nonhuman primates [9,10]. However, the human disease has not been fully replicated in any animal model, and spontaneous fragility fractures resulting from minimal trauma appear to be rare outside of non-human primates [11,12].

Osteoporotic fractures are common in the hip, pelvis, spine, distal radius, and proximal humerus [13,14,15], resulting in pain, hospitalization, height loss, and deformity. Patients often require rehabilitation and experience impaired quality of life due to loss of independence and restricted mobility [14,15]. Hip fracture is the leading cause of emergency surgery in older adults [13]. Beyond pain and disability, fractures, particularly hip and vertebral fractures, are also associated with substantial mortality risk, with approximately 20–30% of patients dying within one year following a hip fracture [14,15,16,17].

A review including 86 international studies estimated the worldwide prevalence of osteoporosis, as defined by the World Health Organization (WHO) diagnostic criteria, as 18.3%, with a marked gender bias: 23.1% in women and 11.7% in men. In 2019, there were an estimated 178 million incident fractures worldwide, 33% more than in 1990, representing an estimated prevalence of 455 million fractures, a 70% increase since 1990 [18]. In Europe, in 2019, it was estimated, according to WHO diagnostic criteria, that 25.5 million women and 6.5 million men suffered from osteoporosis. Also in this year, osteoporosis, together with the 4.3 million new fragility fractures reported, accounted for an estimated economic burden of approximately €56.9 billion, which was expected to increase by approximately 25% by 2025 [15,19]. Between 2010 and 2019, the population of women aged ≥50 in the European Union (EU) 27 + 2 increased by 22%, and that of men by 17%. Both populations are expected to continue to increase by 10% and 13%, respectively, between 2019 and 2034. Furthermore, around 71% of older women at high risk of fracture do not receive adequate assessment or treatment, reflecting a significant gap in osteoporosis management [1,15].

In Spain, an estimated 2.9 million people had osteoporosis in 2019 (2.3 million women and 0.6 million men aged >50 years) [15]. Thus, the prevalence in the total population was 5.4% (22.6% in women and 6.8% in men) [15]. This resulted in 285,494 new fractures, with an associated cost of €4.3 billion, representing 3.8% of national healthcare expenditure. Population projections for 2019–2034 estimate increases of 17.9% in men and 20.3% in women aged 50–74 years, and of 37.7% in men and 28.6% in women aged ≥75 years [15].

Despite the high burden of osteoporosis and its associated fractures, a significant treatment gap persists globally [1,15]. Optimizing bone health through adequate calcium and vitamin D intake is fundamental, as these nutrients are essential for bone mineralization and maintenance, especially considering that vitamin D deficiency remains highly prevalent even among patients with osteoporosis [20,21]. Thus, effective preventive and therapeutic strategies addressing these nutritional deficiencies are essential [3,14,22]. Thus, preventive supplementation aims to reduce future bone loss, falls, and fractures, whereas therapeutic supplementation aims to correct calcium and/or vitamin D insufficiency in patients with established osteoporosis, particularly when used as an adjunct to anti-osteoporotic pharmacological therapy to optimize efficacy and safety [3,13,23,24].

This narrative review summarizes the scientific evidence on the role of combined calcium and vitamin D supplementation in the prevention and treatment of osteoporosis. It addresses the pathophysiological basis, definition, and prevalence of vitamin D deficiency, the results of clinical studies, comparisons of vitamin D doses and formulations, recent guidelines, and practical recommendations for prescribing in clinical practice.

## 2. Methods

This narrative review was based on a literature search to identify relevant evidence and clinical guidance on the role of combined calcium and vitamin D supplementation in osteoporosis. Searches were conducted in PubMed/MEDLINE using combinations of the following terms: “osteoporosis”, “postmenopausal osteoporosis”, “calcium supplementation”, “vitamin D supplementation”, “combined calcium and vitamin D”, “cholecalciferol”, “supplementation”, “dietary intake” “fracture”, “fracture prevention” “falls”, “bone mineral density”, “bone health”, “antiresorptive therapy”, “anabolic therapy”, “treatment adherence” and “clinical guidelines”. Additional references were identified by manually reviewing the reference lists of selected articles and relevant clinical practice guidelines.

The articles were selected based on their relevance and the objectives of the review, with priority given to clinical practice guidelines, consensus statements, randomized controlled trials, systematic reviews, meta-analyses, observational studies with clinical relevance, and health economics analyses. Publications were considered when they addressed calcium and/or vitamin D supplementation in relation to osteoporosis, fracture prevention, falls, bone mineral health, or the optimization of pharmacological treatment for osteoporosis. Articles not focused on osteoporosis or fracture risk, duplicates, and publications with limited relevance to the scope of the review were excluded. Both recent publications and seminal studies were considered when relevant to the discussion. Articles published in English or Spanish were considered.

Given the narrative nature of this review, no formal systematic review protocol, quantitative synthesis, or structured risk-of-bias assessment was performed.

## 3. Calcium and Vitamin D in Bone Metabolism: Mechanisms of Action, Epidemiology and Clinical Implications

Bone metabolism involves a dynamic balance between formation and resorption, processes that depend on adequate calcium availability and the coordinated actions of vitamin D and parathyroid hormone (PTH). Approximately 99% of the body’s calcium is found in bones, where it provides structural support and is stored [25]. The remaining 1% participates in vital functions, including nerve transmission, muscle and vascular contraction, enzymatic and hormonal activity, and transmembrane transport [25]. Thus, bone tissue acts as a calcium reservoir to ensure the efficiency of these physiological processes, regulating its release from bone through bone remodeling [26].

Vitamin D is a fat-soluble steroid hormone precursor of 1,25-dihydroxyvitamin D, essential for bone mineralization and the prevention of osteomalacia. It is synthesized in the skin after exposure to sunlight, a process that begins when ultraviolet B (UV-B) radiation (wavelengths of approximately 280–310 nm) acts on 7-dehydrocholesterol (provitamin D), triggering its photochemical conversion [27,28,29]. Calcitriol [1,25(OH)2D], the active metabolite of vitamin D, acts principally through the vitamin D receptor (VDR), which is expressed in most tissues throughout the body [23,27,30]. The vitamin D endocrine system, through calcitriol, regulates calcium and phosphorus homeostasis and ensures proper bone mineralization. It acts on four organs: in the intestine, increasing the absorption of both minerals; in the kidney, promoting their tubular reabsorption; in the parathyroid, suppressing PTH secretion; and in the bone, modulating the differentiation of osteoclasts and osteoblasts, as well as the synthesis of key proteins for mineralization, such as osteopontin and osteocalcin [23,26,31].

The interplay between calcium and vitamin D determines bone health through distinct pathophysiological mechanisms. When vitamin D is deficient, intestinal calcium absorption decreases significantly, leading to reduced ionic calcium levels. This reduction is detected by calcium sensors in the parathyroid glands (CaSR), triggering secondary hyperparathyroidism characterized by elevated PTH levels [30,32]. Elevated PTH levels increase bone turnover, promote greater resorption than formation, enhance renal tubular reabsorption of calcium, and stimulate renal conversion of 25-hydroxyvitamin D [25(OH)D] to calcitriol [30,33]. On the contrary, when calcium levels are insufficient despite adequate vitamin D status, vitamin D signaling in bone cells helps maintain normocalcemia by increasing bone resorption and reducing mineralization, thus prioritizing serum calcium preservation even at the expense of skeletal integrity [31]. In both scenarios, the skeletal reservoir is mobilized to maintain vital calcium-dependent physiological functions.

Thus, when considering the impact by life stage, recurrent or prolonged periods of vitamin D deficiency can contribute to the risk of osteoporosis by altering calcium absorption, promoting secondary hyperparathyroidism, increasing bone turnover, and compromising bone mineralization [26,34]. Consequently, although osteoporosis is a multifactorial disease, vitamin D deficiency is considered a key and preventable risk factor for its development, even if it is not its sole underlying cause [6,7,8]. Therefore, during growth, insufficient intake of vitamin D and calcium can limit the achievement of optimal peak bone mass [3,35,36]. And in both adults and people with osteoporosis, these mechanisms originated by prolonged vitamin D deficiency can contribute to reduced BMD and increased skeletal fragility [26,34,37]. Insufficient vitamin D also has adverse muscular effects, increasing the risk of falls and, therefore, the likelihood of fractures [33,37]. Furthermore, vitamin D deficiency can have repercussions for overall health, as it affects multiple extraskeletal tissues throughout life, including the cardiovascular and immune systems, and regulates cell differentiation and proliferation [23,31,38].

Serum concentration of 25-hydroxyvitamin D [25(OH)D] is the most appropriate biomarker of vitamin D status [24,27,30]. Severe vitamin D deficiency is defined by 25(OH)D levels < 10 ng/mL (25 nmol/mL), moderate deficiency or insufficiency by values between 10 and 20 ng/mL (25–50 nmol/mL), and concentrations > 30 ng/mL (75 nmol/mL) are considered optimal [30,32]. The threshold of 30 ng/mL for defining vitamin D sufficiency is primarily supported by its association with optimal suppression of PTH [39]. According to published studies, in adults aged ≥65 years, maximal PTH suppression is achieved with 25(OH)D concentrations of approximately 72 nmol/L (equivalent to 30 ng/mL) [39,40].

Vitamin D deficiency is a major global health problem. A systematic review and pooled analysis of 308 population-based studies including 7.9 million participants from 81 countries (2000–2022) found that globally, 47.9% had serum 25(OH)D levels < 50 nmol/L (deficiency) and 76.6% had levels < 75 nmol/L (insufficiency), with higher rates in high-latitude regions, the Eastern Mediterranean, and lower-middle-income countries [41]. Estimated deficiency rates exhibit marked geographical heterogeneity, with severe deficiency (<25 nmol/L) having a prevalence of 5–18% in Latin America, North America, and Oceania, compared to 24–49% in Europe, Africa, and Asia [42]. Additionally, individuals with highly pigmented skin have lower 25(OH)D concentrations, as observed in studies conducted in the United Kingdom and Finland [24].

This global variability cannot be explained solely by latitude or sun exposure. Vitamin D status should be interpreted as a dynamic variable, rather than a fixed biological characteristic, since serum 25(OH)D concentrations vary throughout the year, generally reaching their lowest levels in late winter or early spring and increasing after periods of greater sun exposure [27]. Therefore, it is important to note that a single measurement of 25(OH)D reflects vitamin D status at a specific point in time and does not fully capture recurrent or prolonged periods of insufficiency [24,27]. Seasonal variation, latitude, time spent outdoors, skin pigmentation, clothing habits, sunscreen use, age, obesity, and outdoor physical activity can influence 25(OH)D levels over time [23,27,43]. In fact, regular exercise, especially outdoors, can increase sun exposure and is associated with higher 25(OH)D levels, whereas a sedentary lifestyle and reduced mobility increase the risk of deficiency [3,27,44]. Thus, recurrent or sustained hypovitaminosis D is more significant than an isolated low value, as prolonged impairment of calcium absorption can promote secondary hyperparathyroidism, increase bone turnover, compromise mineralization, and contribute to skeletal fragility [26,34]. In this case, among the various factors that play a role in this global deficiency, such as access to fortified foods, skin type, age, obesity, wearing clothing that limits sun exposure, and the presence of clinical disorders that alter vitamin absorption or metabolism, dietary factors such as vitamin D intake also play a role [27]. Moreover, because vitamin D is essential for intestinal calcium absorption and plays a crucial role in maintaining normal blood calcium levels and bone mineralization, vitamin D and calcium deficiencies often co-occur [25]. It is estimated that nearly half of the world’s population has inadequate calcium dietary intake. Calcium is essential for bone health, and insufficient intake has been linked to rickets, osteomalacia, osteopenia, osteoporosis, and fractures, as well as other systemic consequences [25].

## 4. Clinical Evidence of the Combined Calcium and Vitamin D Supplementation on Fractures

Osteoporosis and fragility fractures place a significant burden on healthcare systems and impair patients’ quality of life, associated with morbidity and mortality. Thus, preventing osteoporotic fractures is crucial in an aging global population. Given the frequent coexistence of low calcium intake and hypovitaminosis D, the use of calcium and vitamin D supplementation as a preventive strategy in people with insufficient intake or at high risk has been investigated to reduce fracture risk.

From a biological perspective, combined supplementation is generally preferable to vitamin D alone, since vitamin D promotes intestinal calcium absorption but, if calcium intake is insufficient, it may not provide the necessary substrate for bone mineralization [27,45,46,47]. Thus, combined supplementation corrects vitamin D deficiency and ensures adequate calcium availability, which is especially relevant for patients with osteoporosis, increased fracture risk, or impaired calcium–vitamin D homeostasis [3,39,46].

In 2007, a meta-analysis of nine randomized clinical trials, which included 53,260 patients, concluded that the combination of calcium and vitamin D supplementation reduced the risk of non-vertebral fracture by 23% (95% Confidence Interval (CI): 1–40) and the risk of hip fracture by 25% (95% CI: 4–42) compared to supplementation with vitamin D alone [45]. A 2014 Cochrane review, based on high-quality evidence, found that calcium plus vitamin D significantly reduced the risk of hip fracture (9 trials, 49,853 participants; risk ratio (RR) 0.84, 95% CI 0.74–0.96; *p* = 0.01) and non-vertebral fractures, though no significant reduction in vertebral fractures was observed. [48]. In 2016, another meta-analysis that included eight randomized controlled trials with a total of 30,970 participants concluded that patients with calcium plus vitamin D supplementation presented a statistically significant 30% reduced risk of hip fractures Summary Relative Risk Estimate (SRRE), 0.70; 95% CI, 0.56–0.87) and a 15% reduced risk of total fractures (SRRE, 0.85; 95% confidence interval [CI], 0.73–0.98) [49]. More recently, a 2020 systematic review and meta-analysis of 10 randomized trials established that the combination of calcium and vitamin D reduces the risk of total fractures (RR: 0.74) and hip fractures (RR: 0.61) [50]. Other meta-analyses have also reported the beneficial effect of combined supplementation on the reduction of hip and non-vertebral fractures, and possibly also on vertebral fractures [47,51,52]. However, it is important to note that this evidence includes heterogeneous populations, with differences in age, environment, baseline fracture risk, supplement use, adherence, calcium intake, vitamin D dosage, and the availability of baseline or endpoint 25(OH)D levels. Therefore, these overall estimates of fracture reduction should be interpreted as averages across mixed populations that include individuals who may or may not have had calcium and/or vitamin D deficiency at baseline [47,49,51,52,53].

The benefit seems to depend on the patient’s baseline fracture risk and nutritional status. Greater effects have been reported in underweight individuals, older adults, participants at higher risk of fractures, institutionalized patients, and those with insufficient calcium intake [50]. In clinical practice, this is also consistent with the groups in whom vitamin D supplementation is most often considered, including patients with comorbidities or conditions that impair vitamin D/calcium homeostasis, such as diabetes, chronic kidney disease, parathyroid disorders, liver disease, gastrointestinal malabsorption, obesity, darker skin phototypes, use of medications affecting vitamin D metabolism, and advanced age [14,27]. On the contrary, calcium and vitamin D supplementation seems to provide limited benefits in populations without nutritional deficiency or low baseline risk. Accordingly, a Cochrane study concluded that there is no evidence that calcium and vitamin D supplementation in healthy premenopausal women provides significant benefits for BMD or fracture prevention [22]. Other studies have confirmed that calcium and vitamin D supplementation have no effect in patients without prior hypovitaminosis [54].

The available evidence suggests that the dose and frequency of vitamin D administration significantly influence clinical efficacy. Vitamin D supplementation should be sufficient to ensure that serum 25(OH)D levels reach the threshold level and provide the desired benefits. The amount of vitamin D required to achieve this level also varies according to clinical conditions [55]. Additionally, it is worth noting that the lower the serum 25(OH)D levels, the greater the increase observed after supplementation [30]. Thus, for every 40 International Units (IU)/day of vitamin D_3_ administered orally, there is an approximate average increase of 0.48 ng/mL of 25(OH)D when previous vitamin D values were low. However, this increase, with the same dose, is 0.28 ng/mL when previous 25(OH)D levels are above 28 ng/mL [30]. The 400 IU/day dose was insufficient to detect an effect on fracture rates, which may explain the negative results reported by some studies [56,57]. In contrast, doses of 700–800 IU/day or higher show beneficial effects, and both daily and quarterly (100,000 IU) administration have demonstrated antifracture efficacy [57]. Annual intramuscular administration, however, has shown inconsistent results [58], with existing meta-analyses suggesting no evidence of the effectiveness of intermittent high-dose vitamin D administration at longer intervals in reducing fracture and fall rates [59,60].

Vitamin D may help reduce falls by supporting neuromuscular function, including balance, muscle strength, and postural control, all of which are key contributors to fall risk in older adults [3,23]. The administration regimen and dose of vitamin D, as well as the target population, are also important factors in reducing falls, although the evidence for fall reduction is less consistent than that for fracture reduction. In addition, an increase in falls was observed in older people receiving high, intermittent doses [59,60]. In 2004, a meta-analyses by Bischoff-Ferrari established that daily doses of 700–1000 IU of vitamin D can reduce falls by 19–26% [57]. They also confirmed this benefit in institutionalized older adults, with a 49% reduction achieved through combination therapy [61]. However, studies using high intermittent doses have shown unfavorable results. Sanders et al. observed that an annual bolus dose of 500,000 IU paradoxically increased the risk of falls by 15% and the risk of fractures by 26% [59,60]. Similarly, a study in 2016 reported a higher incidence of falls with monthly doses of 60,000 IU compared with lower dosing regimens [60]. Finally, the VITAL trial showed that supplementation does not prevent falls in healthy adults without a prior deficiency [62].

Overall, the available evidence suggests that daily oral cholecalciferol may be the preferred strategy for routine supplementation. In recent studies, daily doses of 2000–4800 IU maintained adequate 25(OH)D levels throughout treatment, while 800 IU/day proved insufficient in several trials and 1000 IU/day showed mixed results [63]. Both recent studies and various meta-analyses suggest that more frequent administration regimens offer superior benefits in both skeletal and extraskeletal areas [31,51,54,64]. A randomized pharmacokinetic trial comparing daily, weekly, and biweekly regimens with similar cumulative doses showed that the 20 ng/mL threshold was reached fastest with daily dosing (within 2 weeks), producing the highest serum 25(OH)D concentrations and systemic exposure [65]. This is consistent with the DOSTEO real-world study, in which daily calcium 600 mg/cholecalciferol 2000 IU for at least 24 weeks increased 25(OH)D levels in 94.4% of patients and enabled more than 73% to achieve ≥30 ng/mL, while reducing secondary hyperparathyroidism without clinically relevant changes in calcium or phosphate [66]. Daily administration may also offer biological benefits, as it appears to result in less breakdown of vitamin D and maintain more stable 25(OH)D levels than intermittent single-dose regimens [27]. Therefore, daily oral cholecalciferol at guideline-recommended doses (600–2000 IU/day) appears to be the most practical and physiologically sound approach to routine supplementation, whereas in cases such as intestinal malabsorption, parenteral administration would be preferable [3,13,27,35,63,67,68,69,70].

Vitamin D supplementation must be accompanied by adequate calcium intake. Current guidelines recommend a total calcium intake (dietary plus supplemental) of 800–1000 mg/day for the general adult population and 1000–1200 mg/day in older adults and postmenopausal women [3,13,14,35,67,71,72].

Accordingly, the patient groups most likely to benefit from combined supplementation are those with osteoporosis or high fracture risk, together with clinical or nutritional factors that predispose calcium or vitamin D insufficiency, especially given the frequent coexistence of low levels of calcium and vitamin D. Several patient groups may benefit from this supplementation, including those with comorbidities such as diabetes, chronic kidney disease, parathyroid disorders, liver disease, and gastrointestinal malabsorption syndromes [14,27]. People with higher skin phototypes, obesity, or taking medications that affect vitamin D metabolism (anticonvulsants, glucocorticoids, and antiretroviral drugs) may also require vitamin D supplementation [14,27]. Older adults are at particular risk of fracture due to age. Age is an important risk factor, mainly because it is accompanied by decreased calcium and vitamin D intake, reduced skin synthesis of vitamin D, decreased exposure to sunlight, and reduced intestinal and renal tubular absorption and reabsorption of calcium [73,74,75]. From this perspective, we could then distinguish the use of combined supplementation in primary and secondary osteoporosis. Thus, combined supplementation in primary osteoporosis is mainly aimed to correct insufficient intake or low vitamin D status in older adults, postmenopausal women, frail or institutionalized patients, and individuals at high fracture risk [3,35,45,47,67]. In secondary osteoporosis, supplementation should be considered when the underlying condition or its treatment predisposes to calcium or vitamin D insufficiency, such as glucocorticoid exposure, malabsorption, chronic kidney or liver disease, parathyroid disorders, obesity, or drugs affecting vitamin D metabolism [3,13,23,27,35,67,71]. In these cases, moreover, the thresholds for pharmacological intervention may vary, as they are often set at a higher bone mineral density than in postmenopausal osteoporosis [76]. Nonetheless, in both cases, supplementation is always recommended as an adjunct treatment to anti-osteoporotic treatment.

## 5. Calcium and Vitamin D Supplementation in Combination with Antiresorptive Agents and Other Osteoporosis Drugs

Pharmacological treatments for osteoporosis are classified as antiresorptive or anabolic. Antiresorptive drugs (denosumab and bisphosphonates), which are the most commonly used, reduce the risk of fractures by decreasing bone resorption and preserving bone mineral density, as well as stabilizing bone microarchitecture by inhibiting osteoclastic activity [3]. On the other hand, anabolic drugs (e.g., teriparatide, romosozumab) increase bone density and strength by predominantly promoting osteoblast formation, with variable effects on bone resorption [13]. Antiresorptive and bone-forming drugs can cause hypocalcemia due to their inhibitory effect on osteoclasts [34]. They may also be associated with hypovitaminosis D, which is known to be highly prevalent in osteoporotic patients [26,34,37]. Concomitant calcium and vitamin D supplementation is therefore recommended with both antiresorptive and anabolic treatments.

### 5.1. Bisphosphonates

Bisphosphonates are synthetic analogs of pyrophosphate, a natural substance that inhibits bone mineralization [3,14,77]. Alendronate is the bisphosphonate with the most clinical experience [3]. Several studies have demonstrated that the anti-osteoporotic effect of alendronate, combined with calcium and vitamin D supplementation, is superior to that observed with alendronate alone. Thus, improvements in BMD were observed [37,78,79,80,81,82], with higher vitamin D doses (≥1000 IU/day) resulting in greater improvements [80].

Some studies have shown that risedronate, when combined with vitamin D supplementation, leads to a significant improvement in serum 25(OH)D levels and reductions in bone turnover markers and PTH levels in patients with osteoporosis [83,84].

Ibandronate combined with vitamin D and calcium supplementation reduces vertebral fractures by 49% in postmenopausal women with osteoporosis and a history of previous fractures. However, it has not demonstrated efficacy in reducing hip fractures, other non-vertebral fractures, or vertebral fractures in women without prior fractures [3]. When combined with vitamin D, ibandronate decreases bone turnover markers and PTH levels, and increases lumbar and hip BMD [85,86].

Zoledronic acid reduces the risk of vertebral fracture by 70% compared to placebo (RR 0.3, 95% CI 0.24–0.38) and by 41% for hip fractures in combination with vitamin D and calcium supplementation, making it also indicated for the prevention of new clinical fractures in patients who have recently suffered a fragility hip fracture [3,14,87]. However, although zoledronic acid improves BMD in frail elderly women in the spine and total hip, an increase in fractures, falls, and mortality has been observed in this population [14,88], and its use may not be appropriate in this subgroup.

Across multiple studies, bisphosphonate therapy combined with vitamin D supplementation was associated with a reduction or no increase in PTH serum levels [78,89,90,91], reduced bone turnover markers [79,90] or did not negatively affect them [91], and an improvement of the 25(OH)D levels [79,89,90,91]. Thus, many studies emphasize the need to reverse hypovitaminosis to achieve an optimal response to treatment [37,79], underscoring the importance of maintaining 25(OH)D levels ≥30 ng/mL for adequate response to bisphosphonate treatment [37].

### 5.2. Denosumab

Denosumab is a human monoclonal antibody (immunoglobulin G2 [IgG_2_]) that targets and binds with high affinity and specificity to the receptor activator of nuclear factor κB ligand (RANKL) [3,14]. Compared with denosumab monotherapy in the FREEDOM study [3,92], the combination of denosumab with calcium and vitamin D supplementation resulted in a significantly greater increase in BMD [93,94,95,96], especially in the hip [93,94]. The addition of supplements helped stabilize serum calcium levels [96] and inhibit increases in PTH [93,96], crucial for preventing drug-induced hypocalcemia. In addition to preventing hypocalcemia, combination therapy suppressed bone resorption markers at specific time points [96]. Thus, it could be concluded that calcium and vitamin D supplementation play an important role in optimizing the therapeutic benefits of denosumab and maximizing BMD gains in this population. Furthermore, because some studies have reported a hypocalcaemic response to denosumab treatment [97], calcium and vitamin D supplementation is essential for maintaining optimal levels and preventing this effect.

### 5.3. Anabolic Drugs

Teriparatide (rhPTH(1–34)) is the active fragment (1–34) of endogenous human parathyroid hormone [3,14]. Since it appears to deplete 25(OH)D reserves, as observed during teriparatide therapy, where 25(OH)D levels may decrease by ~10–20% after 12 months of treatment in women and men with osteoporosis [98], it is recommended to correct any hypocalcaemia or hypovitaminosis D before and during anabolic treatment to ensure adequate stores of 25(OH)D (≥30 ng/mL) and available calcium, which would maximize induced bone formation and minimize metabolic risks [13,67].

### 5.4. Calcium and Vitamin D Supplementation in Clinical Practice

In the therapeutic setting, calcium and vitamin D supplementation is a fundamental supportive intervention in the management and treatment of osteoporosis. Its role is to optimize bone mineralization and the efficacy and safety of pharmacological treatment by correcting calcium/vitamin D deficiency, maintaining calcium-phosphate homeostasis, reducing secondary hyperparathyroidism, supporting BMD response to anti-osteoporotic treatment, and reducing the risk of fracture in selected high-risk patients. Clinical guidelines emphasize the importance of ensuring adequate intake of these micronutrients through diet and, if the diet is insufficient, through supplements, in any therapeutic plan for osteoporosis [3,13,14,67,99]. Furthermore, supplementation is recommended for patients undergoing pharmacological treatment, given that most studies conducted with these treatments also include supplementation [26], and that many studies have shown that supplementation improves their effect in terms of demonstrating anti-fracture efficacy [37,78,79,80,81,82,85,93,94,95,96]. Additionally, the technical data sheets for the antiresorptive treatment clearly indicate that patients should receive calcium and vitamin D supplementation concomitantly with the antiresorptive treatment [100,101,102,103].

## 6. Comparing Vitamin D Forms and Dosages

Currently, various metabolites are available (Table 1) for treating vitamin D deficiency, differing in potency to increase plasma 25(OH)D levels, half-life, and onset of action [27].

The main oral forms of vitamin D supplementation are cholecalciferol (vitamin D_3_) and ergocalciferol (vitamin D_2_), both fat-soluble vitamins absorbed in the small intestine [27]. However, the two forms are not the same, and several studies have shown that D_3_ is superior to D_2_ in increasing 25(OH)D levels [27,104,105] and in regulating PTH levels [105]. Cholecalciferol is the most common naturally occurring form of vitamin D, as it is the form synthesized in mammalian skin [106]. In addition, the presence of two circulating forms of 25(OH)D, 25(OH)D_3_ and 25(OH)D_2_, adds additional difficulties for methods of detecting serum 25(OH)D levels, as the antibodies used in immunoassays may not detect 25(OH)D_2_ and 25(OH)D_3_ equally. In fact, some studies have observed that the presence of significant amounts of 25(OH)D_2_ can lead to an overestimation or underestimation of total 25(OH)D levels [27,107]. Additionally, the administration of ergocalciferol as a bolus may negatively impact vitamin D metabolism (increasing 24-hydroxylase activity) [108] and be associated with poorer clinical outcomes (increased fall risk) [59].

As for the other existing metabolites, cholecalciferol and calcifediol/calcidiol are used to treat deficiency disorders, while calcitriol and alfacalcidol are used for populations with specific disorders [3,23,35]. In general, both cholecalciferol and calcifediol are effective and safe options for preventing and treating vitamin D deficiency in different populations. However, cholecalciferol is the form most commonly used in the clinical trials and meta-analyses reviewed, and thus, the one with more positive scientific evidence regarding its effects, being its administration the most recommended in clinical guidelines on the management of osteoporosis [23,26,34,35], even when there is no prior determination of vitamin D levels, according to the Spanish Society for Bone and Mineral Metabolism Research (SEIOMM) [23] and the Spanish Society of Geriatrics and Gerontology (SEGG) [43]. Cholecalciferol can be used on a weekly, fortnightly, or monthly basis at equivalent doses, allowing precise dosing in terms of the number of IU administered [26,34], since cholecalciferol is the gold standard for IU equivalence to molecular mass [106].

Calcifediol, an intermediate metabolite between cholecalciferol and calcitriol, has more hydrophilic properties than cholecalciferol, which translates into rapid absorption through the portal system, less sequestration in adipose tissue, lower distribution volume, and a shorter half-life. These characteristics allow for rapid, predictable increases in 25(OH)D and effective suppression of PTH [109]. 25(OH)D derived from calcifediol is distributed more evenly between muscle, circulation, fat, and other tissues [110], and its management is easier, for example, in situations of toxicity [109]. All these characteristics make calcifediol ideal for specific clinical conditions, such as hepatic failure, patients taking medications that could affect cytochrome enzyme activity, patients with obesity, or those with inactivating mutations in genes encoding CYP2R1 [3,27,67]. This distinction is particularly relevant in secondary osteoporosis, where the choice of vitamin D form and route may need to be adapted to the underlying cause of deficiency or altered metabolism. While oral cholecalciferol remains the preferred routine option in most patients with primary osteoporosis, calcifediol or alternative routes may be considered in selected patients [23,27]. However, this rapid increase in 25(OH)D observed with commonly used doses, especially with intermittent regimens, may lead to supraphysiological levels of 25(OH)D [111,112]. This could increase the likelihood of vitamin D toxicity due to prescribing errors [106], which is less likely with cholecalciferol [113]. Thus, when supplementing with calcifediol, frequent monitoring of serum 25(OH)D levels is necessary. Cholecalciferol is stored mainly in fat (with a half-life of approximately 2 months), allowing gradual release and maintaining 25(OH)D levels between 30 and 50 ng/mL more effectively [114].

Calcitriol, the active hormonal form of vitamin D, increases intestinal calcium absorption and reduces PTH secretion. It has a short half-life, requiring daily administration and close clinical supervision. Its use increases the degradation of 25(OH)D, rendering serum 25(OH)D an unreliable marker of adequate vitamin D supplementation [27]. On the other hand, alfacalcidol is a synthetic analog of calcitriol that is converted into it after hepatic 25-hydroxylation, although it is slightly less potent than calcitriol [14]. Both are associated with adverse effects, such as hypercalcemia and hypercalciuria, thus needing monitoring of calcium and phosphate levels [14,27,115]. For safety reasons, calcitrol use is reserved for patients with renal 1-α-hydroxylase deficiency, who are unable to produce calcitriol adequately [27,115,116]. In these cases, dietary calcium supplementation should be avoided or used with caution [14].

### Toxicity of Vitamin D

Vitamin D toxicity (hypervitaminosis D) is a rare but serious condition characterized by concentrations > 150 ng/mL or > 375 nmol/L of calcifediol, and low or undetectable levels of PTH, causing severe hypercalcemia, and potentially leading to complications in the cardiovascular, renal, gastrointestinal, neurological, and musculoskeletal systems [27,117]. Its prevalence is low due to the broad therapeutic index of vitamin D, and most cases of toxicity result from uncontrolled, excessive doses. The level considered to have “no observed adverse effects” is 10,000 IU per day, while the threshold at which adverse effects begin to be detected is 40,000 IU per day [117,118]. Although the exact dose that causes toxicity is unknown, it is likely to be much higher.

Although there is no conclusive scientific evidence establishing a consensus on the recommended daily doses of cholecalciferol for the treatment of osteoporosis, over the last 30 years, the doses of vitamin D used in studies have increased from 250 IU/day [119] to an average of 2500 IU/day [120]. Other studies have proven that these higher doses are safe for patients. Fassio et al. [65] used up to 10,000 IU/day without observing toxicity or serious adverse effects. On the other hand, in the DOSTEO study, a combination of 600 mg calcium (as 1500 mg calcium carbonate) and 2000 IU cholecalciferol in orodispersible tablets was used without toxicity or adverse events and without association with hypercalcemia or hyperphosphatemia [66]. Therefore, vitamin D supplementation, when administered within appropriate ranges under clinical supervision, can be considered safe and effective, with minimal risk of hypervitaminosis D.

## 7. Implications in Clinical Practice

### 7.1. Vitamin D Deficiency and the Use of Calcium and Vitamin D Supplementation in Spain

Considering the estimated average requirement for vitamin D, which ranges from 800 to 2000 IU/day (20–50 μg/day) depending on personal needs, average intake values in Europe are very low: 2.7 for females and 3.3 μg/day for males, with clear regional differences [42]. In Spain, the ANIBES study confirmed an average vitamin D intake of 4.4 µg/day (3.65 µg/day for women and 4.28 µg/day for men), indicating that 94% of the population did not reach 80% of the recommended intake [44]. In addition, although guidelines recommend a daily calcium intake between 700 and 1200 mg/day for adults [3,14,35,67,71,72], the ANIBES study reported an average calcium intake of 698 mg/day, with 76% of the population consuming less than 80% of the recommended daily intake [44].

Despite the high prevalence of vitamin D and calcium deficiency, and the recommendation by clinical practice guidelines that osteoporosis treatments, especially antiresorptive agents, be accompanied by calcium and vitamin D supplementation [3,13,14,67,99], there remains considerable room for improvement in the proportion of patients who actually receive supplementation alongside antiresorptive therapy. In 2012, Carbonell et al. [121] reported that 62.6% of patients with osteoporosis receiving antiresorptive treatment also received supplementation. This percentage was significantly higher in bone metabolism units (UMO)/rheumatology (89.1%) than in primary care (60.3%) and gynecology (55.6%). The survey also revealed that 72% of UMO/rheumatology physicians considered vitamin D supplementation necessary, compared to 38.5% of primary care physicians. Currently, it appears that doctors’ awareness has improved. A recent Delphi consensus conducted by a multidisciplinary panel found that 80.3% of responders supported the supplementation in combination with osteoporotic treatment [122]. However, these percentages were not stratified by specialists, limiting direct comparison with earlier data. Notably, 82.9% of respondents reported a lack of awareness or interest in osteoporosis in primary care, suggesting that implementation of guideline-based calcium and vitamin D supplementation remains an area for improvement, particularly outside specialist settings.

### 7.2. Role of Primary Care in the Management of Patients with Osteoporosis

Primary care plays a key role in fracture prevention because family physicians are responsible for identifying patients at risk, initiating or implementing osteoporosis medication, and ensuring follow-up. The PREFRAOS study [123] reported a prevalence of fragility fractures of 17% among individuals aged ≥70 years attending primary care (24.1% women and 8.0% men), with significant gaps in the diagnosis (two-thirds had an osteoporosis diagnosis) and treatment of osteoporosis (56.8% were not receiving osteoporosis medication).

Family physicians face several barriers to implementing fracture prevention strategies, including unclear or poorly adapted clinical guidelines, high workload and limited resource availability, and the influence of the private sector, which can lead to inaccurate diagnoses and unnecessary treatments [124].

In addition, although a recent Delphi consensus [121] revealed that 98.7% of experts agreed on the need to stratify patients according to fracture risk, and FRAX was considered a helpful tool by 68.7% of participants, fracture risk assessment and screening practices remain heterogeneous among Spanish family physicians [122,124]. This variability may contribute to the low uptake of drug treatment for the primary prevention of fractures, despite the fact that guidelines recommend treating patients at high risk of fracture, even if they have not previously suffered any fractures. Overall, these findings highlight persistent opportunities to improve osteoporosis diagnosis, risk stratification, and evidence-based management in primary care [123,124].

### 7.3. Patient Factors, Adherence, and Persistence to Treatment

Adherence and persistence with osteoporosis treatment are essential for its clinical effectiveness and cost-effectiveness. Optimal adherence should be at least 50% and ideally >70% [122]. However, studies indicate that more than half of patients discontinue treatment within the first year [125]. Low adherence to treatment is linked to suboptimal clinical outcomes and increased risk of fractures [14]. Factors contributing to reduced adherence include dosing frequency, adverse effects, treatment complexity, and patient-related barriers [3]. A systematic review of 20 studies demonstrated that simplifying dosing regimens, implementing electronic prescribing, and involving pharmacists all improve adherence and persistence with osteoporosis treatment [126]. Furthermore, using pharmaceutical formulations that facilitate administration and monitoring compliance may enhance treatment adherence [3].

Since clinical practice guidelines recommend combining osteoporosis treatment with calcium and vitamin D, and daily supplementation appears to be more effective, fixed-dose combination formulations would be a good approach. Recently, Pinto-Bonilla et al. conducted the DOSTEO study, in which they used daily orodispersible tablets containing 600 mg of calcium and 2000 IU of cholecalciferol [66,127]. The regimen was safe and well-tolerated, achieving adequate serum 25(OH)D levels with no effect in patients who were basally replete, and reducing PTH levels, demonstrating that this combination is both safe and effective [66].

Patient beliefs and understanding also influence adherence. A study involving family physicians reported that postmenopausal women often hold misconceptions about osteoporosis, frequently believing that it causes pain and confusing it with osteoarthritis. This represents a significant issue, particularly regarding their understanding of the role of supplementation and exercise [124]. Improving clinical outcomes requires patient education, personalized treatment, and shared decision-making (SDM), supported by protocols tailored to individual needs and the promotion of healthy lifestyle habits [8,124]. Clinical guidelines recommend incorporating patients’ beliefs, values, and preferences through SDM [128]. Decision aids, such as bone densitometry tests, facilitate physician–patient communication, improve knowledge of risks and benefits, and reduce decisional conflict [124,129]. A lack of time, insufficient institutional support, and the absence of clear policies mainly limit their implementation. However, although their inclusion in clinical guidelines, the use of incentives, and the optimization of electronic systems could promote their integration [124].

The management of patients with osteoporosis should be multidisciplinary [3,122], as exemplified by Fracture Liaison Service (FLS) units, which coordinate the identification, treatment, and follow-up of patients with fragility fractures, thereby reducing the risk of new fractures and improving, among other aspects, treatment adherence [3,14,67,130].

In addition, emphasizing the patient’s lifestyle to help enhance the beneficial effects of supplementation is an important factor. A balanced diet is necessary [3], with increased consumption of foods rich in calcium and vitamin D or fortified foods; avoid tobacco use, which is associated with decreased BMD, an increased risk of fracture, decreased osteoblastic activity in bone, and reduced intestinal calcium absorption [3]; and avoid excessive alcohol consumption, as chronic and heavy drinking suppresses osteoblastic activity and is associated with alterations in the bone mineral metabolism of calcium, phosphorus, and magnesium, in addition to disrupting vitamin D metabolism due to damage to the liver, where this hormone is synthesized [3].

Other patients’ factors may influence the effectiveness of the combined supplementation. Patients with a high Body Mass Index (BMI) have lower vitamin D bioavailability because vitamin D is sequestered by adipose tissue or due to a “volumetric dilution” effect [27,35,36]. They typically require doses that are 2 to 3 times higher to achieve the same serum levels as a person without obesity [3,23]. Medications such as glucocorticoids, antiepileptic drugs, or antiretrovirals can interfere with vitamin D metabolism, requiring higher supplemental doses to be effective [3,23]. Conditions such as celiac disease, inflammatory bowel disease, or bariatric surgery (such as gastric bypass) drastically reduce the absorption of these nutrients, limiting the effectiveness of standard oral supplements [23,27,35,36].

There are also genetic factors, such as polymorphisms and mutations that can influence how each individual metabolizes and responds to supplementation. Variations in genes encoding the VDR, the vitamin D-binding protein (DBP), or enzymes involved in its activation (such as CYP2R1 in the liver and CYP27B1 in the kidney) can alter the bioavailability and hormonal action of vitamin D [27,51]. However, although these factors could explain some of the individual variability in the conversion of supplemental vitamin D to 25(OH)D, they have not yet been fully characterized [3].

Thus, individualization in clinical practice should primarily be based on clinical risk, dietary intake, vitamin D status when available, comorbidities, medication profile, lifestyle factors, adherence, and patient preferences [3,23,27,35,122].

### 7.4. Monitoring of the Patients and Safety Considerations

The detection of vitamin D deficiency and preventive interventions are considered cost-effective and cost-efficient, as low serum 25(OH)D concentrations not only indicate increased fracture risk but are also associated with various diseases [23,31,38]. Although systematic population screening would enable the early identification of individuals who could benefit from vitamin D supplementation before clinical consequences develop, the high cost of routine serum 25(OH)D determination currently renders this approach unjustified [24,27], since the determination of 25(OH)D can cost up to ten times more than several months of vitamin D supplementation [24]. A more efficient approach is to focus screening on populations at high risk of deficiency, such as the elderly, pregnant women, individuals with chronic diseases (such as liver disease and parathyroid disorders), and patients with obesity [27,131].

Osteoporosis treatment monitoring is based on laboratory assessments, clinical evaluation, detection of new fractures, and measurement of BMD by Dual-energy X-ray Absorptiometry (DXA) [67].

Routine monitoring is not necessary in the general population when supplementation is within recommended limits [27]. However, monitoring would be advisable for patients at higher risk of altered calcium–phosphate metabolism or vitamin D toxicity, such as those receiving high-dose supplementation or vitamin D metabolites other than cholecalciferol, patients with malabsorption, chronic kidney disease, parathyroid or liver disease, previous hypervitaminosis D, hypo- or hypercalcemia, hypercalciuria, or hyperphosphatemia [39,70]. Thus, in usual practice, monitoring the effectiveness of combined calcium and vitamin D supplementation should be individualized according to baseline risk, comorbidities, concomitant anti-osteoporotic treatment, and the presence of factors that may alter calcium–phosphate metabolism.

If biochemical monitoring is indicated, serum 25(OH)D is the primary marker of vitamin D status, with a therapeutic target of 30–50 ng/mL [23,35,106]. Spanish guidelines recommend initial follow-up every 3–4 months and, once stable levels are achieved, every 6–12 months [23]. Monitoring of PTH and 24 h urinary calcium is also recommended to assess calcium homeostasis and absorption [35,46,71].

Bone turnover markers, such as N-terminal propeptide of type I procollagen (P1NP) and Carboxy-terminal telopeptide of type I collagen (CTX), allow for early detection of adherence and biological response between 3 and 6 months of treatment [14,35,67], especially in patients receiving anti-osteoporotic pharmacological therapy. Central DXA bone densitometry remains the standard for monitoring bone mass, with follow-up every 1–3 years depending on the patient’s risk [35,36,132]. From a clinical perspective, it is essential to record the incidence of falls and height loss of ≥2 cm, as these may indicate asymptomatic vertebral fractures [3,36,130]. Finally, periodic assessment of adherence is critical, since poor adherence can negate the preventive benefits, and clinical monitoring, including assessment of incident fractures, falls, musculoskeletal symptoms, adverse events, and dietary calcium intake, is necessary [67,122].

It is recommended to remain vigilant for potential adverse effects of supplementation. In the case of calcium, the main concerns relate to its potential effects on the kidneys, the cardiovascular system, and gastrointestinal tolerability. High-dose supplementation has been linked to an increased risk of nephrolithiasis [13,35,67], although the magnitude of this effect appears to depend on the clinical context and baseline dietary intake. The possible association between calcium supplements and cardiovascular events remains a subject of debate [14,35,46,81]. A meta-analysis reported that calcium supplements administered without vitamin D were associated with a 30% increased risk of myocardial infarction [133]. However, this finding is not consistently supported by more recent evidence [14]. Nonetheless, calcium and vitamin D supplementation should be used with caution in patients with cardiovascular disease, as these supplements may potentiate the effects of cardiac glycosides [117].

Additionally, calcium supplementation may cause mild gastrointestinal discomfort, such as constipation, flatulence, nausea, gastric pain, or diarrhea, although these effects do not occur consistently [56,134]. In addition, excessive calcium intake may contribute to hypercalcemia and hypercalciuria [27,39], situations that may require a dose adjustment or discontinuation of supplementation [3,14,35].

Although vitamin D toxicity is rare, it can be severe when associated with marked hypercalcemia [27,30]. Clinical manifestations may include gastrointestinal symptoms, intense thirst, neuropsychiatric abnormalities, and, in cases of prolonged exposure, irreversible renal or cardiovascular damage, including nephrocalcinosis and calcification of soft tissues or blood vessels [27,43]. In addition, very high doses or intermittent bolus doses of vitamin D may have paradoxical effects on musculoskeletal health. Infrequent bolus doses, such as intermittent doses ≥60,000 IU or 500,000 IU per year, have been associated with an increased risk of falls and fractures [27,39,60], possibly due to sudden fluctuations in vitamin D levels or the activation of catabolic pathways [24,35,60]. Similarly, high daily doses could negatively affect bone mineral density, perhaps due to increased bone turnover, elevated FGF23 levels, and induction of 24-hydroxylation, thereby reducing the functional availability of 1,25(OH)_2_D despite elevated levels of 25(OH)D [24].

When calcium and vitamin D are administered together in excessive amounts, the risk of hypercalcemia, hypercalciuria, and nephrolithiasis may increase [13,35]. However, calcium and vitamin D supplementation, when administered according to clinical recommendations, is generally well tolerated and provides clear benefits in the prevention and treatment of osteoporosis [3,27,35].

## 8. Recent Guidelines and Consensus

Healthy lifestyle habits, including an adequate diet, regular physical exercise, fall prevention strategies, and avoidance of harmful habits, constitute the primary recommendations for the general population to maintain calcium levels and prevent vitamin D deficiency and osteoporosis [3]. However, many physicians have observed that postmenopausal women perceive physical activity as a cardiovascular benefit, rather than as a protective factor for bone health [124]. Additionally, the ANIBES study found that dietary vitamin D and calcium intakes are below the daily requirements [44]. Thus, calcium and vitamin D supplementation may represent an important alternative to preserve the population’s bone health.

The Spanish clinical practice guidelines on osteoporosis recommend calcium and vitamin D supplementation [3,67,71], as well as the main international guidelines [13,14,35,68,72]. These recommendations distinguish between preventive strategies, focused on achieving adequate dietary intake and correcting insufficiency in people at risk, and therapeutic strategies, in which calcium and vitamin D are used as adjuncts to pharmacological osteoporosis treatment and to prevent treatment-related metabolic complications. Although there is still no consensus on the ideal dose of vitamin D in the context of osteoporosis, the trend over the past 30 years has been a progressive increase from 400 IU to approximately 2000 IU [26]. In fact, more recent studies suggest that higher doses of vitamin D maintain adequate levels more effectively [63,65,66]. Furthermore, most clinical guidelines recommend cholecalciferol supplementation of up to 2000 IU/day in patients with osteoporosis. This recommendation was first established by the International Osteoporosis Foundation (IOF) in 2010 [135], followed by Sociedad Española de Reumatología (SER) [67], Asociación Española para el Estudio de la Menopausia (AEEM) [3], National Osteoporosis Guideline Group (NOGG) [13], and American Association of Clinical Endocrinologists (AACE) [35]. In adults with serum 25[OH]D levels of 20 to 29 or < 20 ng/mL, the AACE even recommends doses up to 5000 IU/day to achieve a 25(OH)D blood level > 30 ng/mL [35]. Other consensus also recommend vitamin D supplementation at doses of 800–2000 IU/day for adults to maintain adequate vitamin D status [27,69,136]. Table 2 summarizes the vitamin D and calcium supplementation recommendations from the most relevant clinical guidelines.

## 9. Cost-Effectiveness and Implementation Considerations

The economic burden of osteoporosis in the European Union was substantial, estimated at €37 billion in 2010, including pharmacological treatment. Of this total, 66% corresponded to the management of new fractures, 5% to pharmacological prevention, and 29% to long-term post-fracture care. When prevention costs were excluded, hip fractures accounted for 54% of total expenditures. Additionally, when assigning a value of twice the Gross Domestic Product (GDP) per quality-adjusted life years (QALY), the total economic value of QALYs lost that year was estimated at €61.4 billion [14,19]. In 2019, the burden of osteoporosis, associated with 4.3 million reported fragility fractures, generated an estimated economic burden of approximately €56.9 billion, with pharmaceutical treatment accounting for only 3% of the total [15]. In Spain, osteoporosis represents a significant burden on our healthcare system, with nearly 285,494 new fractures in 2019 and an associated cost of approximately €4.3 billion, corresponding to 3.8% of total healthcare expenditure [15].

Weaver et al. conducted a cost–benefit analysis among individuals aged 50 years and older to assess the cost-effectiveness of calcium and vitamin D supplementation for preventing osteoporotic fractures [138]. They estimated that more than 30 million people in the European Union (EU), and nearly 11 million in the United States, suffer from osteoporosis. This corresponds to approximately 3.9 and 2.3 million fractures per year, respectively, with hospital costs exceeding €50 billion and $28 billion. If all these individuals received calcium and vitamin D supplementation, approximately 544,687 fractures per year in the EU and 323,566 in the United States could be prevented, resulting in savings of more than €6.9 billion and $3.9 billion, respectively. The net economic benefit would be estimated at €5,710,277,330 and $3,312,236,252, respectively.

Thus, given that the population aged 50 and over is expected to continue increasing, calcium and vitamin D supplementation, from a preventive perspective, is a highly cost-effective strategy, and its broader use in older adults with osteoporosis or high fracture risk could substantially reduce both the incidence of fractures and the associated healthcare costs.

## 10. Prospects and Gaps

### 10.1. Role of the Real-World Evidence

Although randomized controlled trials (RCTs) have been the gold standard to establish safety and efficacy, they are usually long and costly, and their strict inclusion and exclusion criteria limit their generalizability to real-world populations, often excluding patients with multimorbidity and polypharmacy [139,140,141]. In contrast, real-world evidence (RWE), derived from the analysis of real-world data (RWD), provides crucial information on the benefits, risks, and long-term effectiveness of osteoporosis treatments in routine clinical practice, addressing knowledge gaps left by RCTs [139,140]. RWE has also contributed to regulatory decision-making: it supported the authorization of abaloparatide (Eladynos) in Europe [139,140] and denosumab (Prolia) in China [140] since this real-world evidence addressed regulatory concerns regarding efficacy (non-vertebral fractures) and cardiovascular safety [139,140].

However, observational studies are more prone to confounding, selection bias, missing data, exposure misclassification, and variability across data sources [140,141]. Thus, RWE must adhere to high standards of quality and transparency to be reliable [140]. Therefore several factors are relevant to consider when designing an observational study in osteoporosis [141], including the selection of secondary data sources (administrative databases and electronic medical records), drug pharmacology (crucial for adequately measuring exposure), the method used to measure drug exposure, the measurement of adherence through persistence (duration of treatment before discontinuation) and implementation (proportion of days covered over a specific observation period), the importance of long-term exposure, and patterns of treatment switching.

To assess the validity of RWE in the absence of RCTs, several projects are being conducted. The U.S. Food and Drug Administration (FDA)-funded RCT-DUPLICATE project aims to determine if observational studies approaches can replicate the results obtained from the published clinical trials [140]. Meanwhile, in the EU, the European Medicines Agency (EMA) established DARWIN EU, which aims to promote the exchange of health data to support research, regulatory decision-making, and improvements in healthcare delivery across Europe [139].

It is essential to ensure high standards of quality, transparency, and methodological rigor in the design and analysis of observational studies so that RWE can continue to make a meaningful contribution to regulatory and clinical decision-making, optimizing the care of patients with osteoporosis in real-world clinical practice.

### 10.2. Forms of Supplementation

Calcium and vitamin D supplements are available in various pharmaceutical forms, which is important because the route of administration strongly influences patients’ preferences and adherence to long-term osteoporosis therapy [142]. The most common oral formulations include conventional tablets or capsules, chewable tablets, effervescent sachets (granules to be dissolved in water), liquid solutions, and drops. The availability of different pharmaceutical forms allows the medication to be adjusted to the patient’s preferences.

Additionally, new pharmaceutical forms are being developed, such as bucodispersible forms. These forms dissolve in the patient’s mouth with minimal saliva within a few minutes, without requiring water, and eliminate the risk of choking, thereby helping to overcome swallowing difficulties [143,144].

## 11. Conclusions

This narrative review highlights the critical role of daily calcium and vitamin D supplementation in both fracture prevention and the therapeutic management of osteoporosis. Combined supplementation is more effective than vitamin D alone in reducing the risk of non-vertebral and hip fractures, particularly in older adults, frail or institutionalized individuals, patients with osteoporosis or high fracture risk, and individuals with vitamin D deficiency, insufficient calcium intake, or conditions that impair calcium–vitamin D homeostasis. In the therapeutic setting, adequate calcium and vitamin D status are essential to maximize the efficacy and safety of anti-osteoporotic therapies, such as bisphosphonates and denosumab, which can induce or exacerbate hypocalcemia and hypovitaminosis D. Clinical guidelines recommend individualized regimens, with cholecalciferol (vitamin D_3_) as the preferred form due to its efficacy and safety. Optimal daily intakes are generally 800–2000 IU for vitamin D and 1000–1200 mg for calcium (from diet and supplements). Despite these recommendations, many patients do not receive adequate supplementation when receiving antiresorptive therapy. Improving patients’ adherence, individualizing supplementation regimens, emphasizing patient education, and simplifying administration by using combined formulations could improve clinical outcomes. Finally, broader implementation of calcium and vitamin D supplementation constitutes a cost-effective strategy to reduce fracture incidence, improve quality of life, and decrease the healthcare burden associated with osteoporosis.

## Figures and Tables

**Table 1:** Forms/metabolites of vitamin D.

Vitamin D Form	Key Features	Common Route/Formulations	Advantages	Drawbacks/Precautions
**Cholecalciferol (vitamin D_3_)**	Animal-derived provitamin D_3_. Requires hepatic and renal activation.	Oral (tablets or capsules, alone or with Ca). IM in cases of malabsorption.	Greater anti-fracture evidence. Recommended by most guidelines. Flexible administration schedule (daily/weekly/monthly); safe in the long term. Long half-life (accumulates in adipose tissue)	Slower response in obesity, liver disease, or malabsorption. Avoid annual megabolus.
**Ergocalciferol** **(vitamin D_2_)**	Fungi-based vitamin D2.	Oral, high-dose capsules (50,000 IU) for deficiency correction.	Available in high-dose formulations for deficiency. Alternative for vegetarians.	Less effective than D3 in increasing and maintaining 25(OH)D. Preferring cholecalciferol according to recent consensus.
**Calcifediol/calcidiol (25-hydroxyvitamin D_3_)**	Prehydroxylated vitamin D. Inactive vitamin D metabolite, produced in the liver.	Oral (capsules or solutions). In cases of liver disease, obesity, malabsorption, or alterations of 25-hydroxylation.	Corrects hypovitaminosis D faster. Predictable levels. Less dependent on liver function.	Shorter half-life. Risk of overdose if doses equivalent to D3 are applied. Less evidence of long-term anti-fracture.
**Calcitriol (1,25(OH)_2_D_3_)**	Active form of vitamin D. Acts independently of renal activation.	Oral (capsules). Active drug, not standard supplement.	Useful in severe alterations of vitamin D metabolism.	Risk of hypercalcemia and hypercalciuria. Requires monitoring. Not recommended for routine supplementation. Not for osteoporosis
**Alfacalcidol (1α-hydroxyvitamin D_3_)**		Oral (synthetic analogue). Calcitriol prodrug.	Alternative to calcitriol in specific pathologies.	Risk of hypercalcemia. Not recommended as a regular supplement.

Ca: calcium; 25(OH)D: 25-hydroxyvitamin D; IM: Intramuscular; IU: International Units [14,27,35,67].

**Table 2:** Main clinical practice guidelines recommendations for calcium and vitamin D.

Guideline	Daily Recommended Calcium Intake	Daily Recommended Vitamin D Intake	Use of Antiresorptives with Calcium and Vitamin D	References
**International Osteoporosis Foundation (IOF)**	Post-menopause (51+ years): 1200 mg During pregnancy/lactation (14–18 years):1300 mg During pregnancy/lactation (19–50 years): 1000 mg Men 70+ years: 1200 mg	Generally recommend 800–1000 IU/day for adult population. 2000 IU/day: patients with osteoporosis, individuals with obesity, limited sun exposure (institutionalized, homebound), and malabsorption, and in non-European populations known ≥60 years old: 800–1000 IU/day		International Osteoporosis Foundation [68,72] Dawson-Hughes et al. [135]
**European guidance for the diagnosis and management of osteoporosis in postmenopausal women** **International Osteoporosis Foundation (IOF) and the European Society for Clinical and Economic Aspects of Osteoporosis, Osteoarthritis and Musculoskeletal Diseases (ESCEO)**	800–1000 mg per day in men and women aged ≥50 years 0.5–1.2 g in patients receiving bone protective therapy	800 IU per day in men and women aged ≥50 years 400–800 IU in patients receiving bone protective therapy	The efficacy of interventions is based on co-administration of the agent with calcium and vitamin D supplements.	Kanis et al. [14]
**Evidence-Based Guideline for the** **management of osteoporosis in men** **European Society** **for Clinical and Economic Aspects of Osteoporosis, Osteoarthritis and** **Musculoskeletal Diseases (ESCEO)**	800–1200 mg of calcium/day through diet. If daily calcium intake is less than 800 mg, supplementation may be considered.	800 IU of vitamin D daily for men at higher risk of fractures or with insufficient vitamin D levels.	Correct levels of vitamin D and calcium are essential in treatment.	Fuggle et al. [137]
**NOOG 2024: Clinical Guideline for the Prevention and Treatment of Osteoporosis** **National Osteoporosis Guideline Group (NOGG) (UK)**	700 mg for adults aged ≥ 19 years.	400 IU daily of vitamin D for adults of all ages Osteoporosis patients: 800 up to 2000 IU daily. Routine intermittent administration of large doses of vitamin D, e.g., ≥ 60,000 IU, is not advised.	Treat vitamin D deficiency and insufficiency prior to initiation of anti-osteoporosis drug treatment. Offer calcium and/or vitamin D supplementation as an adjunct to anti-osteoporosis drug treatment, if dietary calcium is low and/or vitamin D insufficiency is a risk, respectively	National Osteoporosis Guideline Group [13]
**Clinical practice guidelines for the diagnosis and treatment of postmenopausal osteoporosis-2020 update** **American Association** **of Clinical Endocrinologists (AACE)**	Total intake (diet plus supplement) of 1200 mg/day for women aged ≥ 50 years. For optimal absorption, calcium supplementation should not exceed 500 to 600 mg per dose.	Adults with vitamin D insufficiency or deficiency (serum 25[OH]D 20 to 29 or < 20 ng/mL, respectively): 5000 IU vitamin D_3_ daily for 8 to 12 weeks to achieve a 25(OH)D blood level > 30 ng/mL, followed by maintenance therapy of 1000 to 2000 IU of vitamin D_3_ daily Supplement with vitamin D_3_ if needed, with a daily dose of 1000 to 2000 IU Higher doses of vitamin D_3_ may be necessary in patients with obesity, malabsorption, and older age	Assess the adequacy of calcium and vitamin D through laboratory evaluation prior to initiation of pharmacologic therapy for osteoporosis.	Camacho et al. [35]
**2019 Recommendations from the Spanish Society of Rheumatology on Osteoporosis** **Spanish Society of Rheumatology (SER)**	Postmenopausal women with osteoporosis: 800–1200 mg/daily Primary prevention: 1000–1200 mg, mainly from the usual diet, for women > 50 and men > 70 1000 mg/day in women aged < 50 and men between 51 and 70. For optimal absorption, calcium supplementation should not exceed 500 mg per dose.	800 IU daily. 800 to 2000 IU/day depending on baseline levels.	-	Naranjo et al. [67]
**Executive summary of the clinical practice guidelines on postmenopausal, glucocorticoid-induced and male osteoporosis (2022 update)** **Spanish Society for Bone Research and Mineral Metabolism (SEIOMM)**	1000–1200 mg/day, preferably through a balanced diet, with supplements as needed.	Generally recommend 800–1200 IU/day (or its weekly or monthly equivalent). If calcifediol is used, 0.266 micrograms every 15–30 days.	Patients treated with osteoporosis drugs should receive adequate calcium and vitamin D to achieve serum 25OHD levels > 25–30 ng/mL.	Riancho et al. [71]
**Osteoporosis MenoGuide** **Spanish Association for the Study of Menopause (AEEM)**	1000–1200 mg through diet. Pharmacological supplementation will only be administered if this intake is less than 700 mg/day. To avoid possibility of adverse events, not routinely exceed 2000 mg/day.	Severe deficiency levels: < 10 ng/mL Cholecalciferol: 1000–2000 IU/day, 25,000–30,000 IU/week, or 50,000 IU/fortnight for 6–8 weeks. (Subsequently, continue with the deficiency regimen). Calcifediol 266 µg/week (16,000 IU/week) for 4–5 weeks. (Subsequently, continue with the insufficiency regimen). Insufficiency levels: 10–30 ng/mL Cholecalciferol: 25,000–30,000 IU/fortnight or 50,000 IU/month for 1–2 months. (Subsequently, 800–1000 IU/day or 25,000–30,000 IU/month).—Calcifediol: 266 µg/month (16,000 IU/month).	Antiresorptive agent accompanied by calcium and vitamin D supplementation. Adequate vitamin D levels are essential for optimal efficacy of antiresorptive treatments.	Presa Lorite et al. [3]

## Data Availability

No new data were created or analyzed in this study. Data sharing is not applicable to this article.

## Abbreviations

The following abbreviations are used in this manuscript:

25(OH)D	25-hydroxyvitamin D
AACE	American Association of Clinical Endocrinologists
AEEM	Asociación Española para el Estudio de la Menopausia
BMD	Bone Mineral Density
BMI	Body Mass Index
CaSR	Calcium-sensing receptor
CI	Confidence Interval
CTX	Carboxy-terminal telopeptide of type I collagen
DBP	Vitamin D-binding protein
DXA	Dual-energy X-ray Absorptiometry
EMA	European Medicines Agency
EU	European Union
FDA	U.S. Food and Drug Administration
FLS	Fracture Liaison Service
FRAX	Fracture Risk Assessment Tool
GDP	Gross Domestic Product
IgG2	Immunoglobulin G2
IOF	International Osteoporosis Foundation
IU	International Units
NOGG	National Osteoporosis Guideline Group
P1NP	N-terminal propeptide of type I procollagen
PTH	Parathyroid Hormone
QALY	Quality-Adjusted Life Years
RANKL	Receptor Activator of Nuclear Factor Κb Ligand
RCTs	Randomized Controlled Trials
RR	Risk ratio
RWD	Real-World Data
RWE	Real-World Evidence
SDM	Shared Decision-Making
SEGG	Spanish Society of Geriatrics and Gerontology
SEIOMM	Spanish Society for Bone and Mineral Metabolism Research
SER	Sociedad Española De Reumatología
SRRE	Summary Relative Risk Estimate
UMO	Bone Metabolism Units
UV-B	Ultraviolet B
VDR	Vitamin D Receptor
WHO	World Health Organization
